# Comparative transcriptomics reveals commonalities and differences in the genetic underpinnings of a floral dimorphism

**DOI:** 10.1038/s41598-022-25132-2

**Published:** 2022-12-01

**Authors:** Giacomo Potente, Rebecca L. Stubbs, Narjes Yousefi, Walter Pirovano, Péter Szövényi, Elena Conti

**Affiliations:** 1 Department of Systematic and Evolutionary Botany, University of Zurich, Zurich, Switzerland; 2 BaseClear BV, Leiden, The Netherlands; 3 Zurich-Basel Plant Science Center, Zurich, Switzerland; 4 Department of Complex Trait Genetics, Center for Neurogenomics and Cognitive Research, Amsterdam Neuroscience, Vrije Universiteit Amsterdam, Amsterdam, The Netherlands

**Keywords:** Evolution, Plant sciences, Plant evolution, Plant reproduction, Genetics, Gene expression, Molecular biology, Transcriptomics

## Abstract

Distyly, a floral dimorphism associated with heteromorphic self-incompatibility and controlled by the *S-*locus supergene, evolved independently multiple times. Comparative analyses of the first transcriptome atlas for the main distyly model, *Primula veris*, with other distylous species produced the following findings. A set of 53 constitutively expressed genes in *P. veris* did not include any of the housekeeping genes commonly used to normalize gene expression in qPCR experiments. The *S-*locus gene *CYP*^*T*^ acquired its role in controlling style elongation via a change in expression profile. Comparison of genes differentially expressed between floral morphs revealed that brassinosteroids and auxin are the main hormones controlling style elongation in *P. veris* and *Fagopyrum esculentum*, respectively. Furthermore, shared biochemical pathways might underlie the expression of distyly in the distantly related *P. veris*, *F. esculentum* and *Turnera subulata*, suggesting a degree of correspondence between evolutionary convergence at phenotypic and molecular levels. Finally, we provide the first evidence supporting the previously proposed hypothesis that distyly supergenes of distantly related species evolved via the recruitment of genes related to the phytochrome-interacting factor (PIF) signaling network. To conclude, this is the first study that discovered homologous genes involved in the control of distyly in distantly related taxa.

## Introduction

Among living organisms, angiosperms (flowering plants) display the highest variability in reproductive organs and mating systems^1^. Distyly is one of the best studied plant mating systems and consists of a floral dimorphism in which male and female sexual organs are reciprocally positioned. Distylous species possess two floral morphs: L-morph (pin) flowers have long style and low anthers, while S-morph (thrum) flowers have short style and high anthers^2^. This main morphological feature is often accompanied by a heteromorphic self-incompatibility mechanism that prevents fertilization between flowers of the same morph^2,3^. Additional ancillary features may further differentiate the two floral morphs^4^, for example number and size of pollen grains^2,5,6^, length and shape of stigma papillae^5,7^, number and shape of cells in the upper corolla tube (i.e. above the anthers’ attachment point), and width of the corolla tube mouth^8^.

Having evolved independently in angiosperms at least 13 times^9^, distyly represents an ideal case to study convergent evolution^10^. Research on distyly has mainly focused on *Primula* (Primulaceae)^11–17^, *Fagopyrum* (Polygonaceae)^18,19^, and *Turnera* (Passifloraceae)^20–23^, reviewed below, and, to a lesser extent, *Linum* (Linaceae)^24^ and *Lithospermum* (Boraginaceae)^25,26^. Phenotypic convergence in floral morphology appears to be mirrored by convergence in the genetic architecture of the locus controlling distyly. Specifically, in all studied species, distyly is controlled by a set of genes clustered together in the same genomic region, forming the so-called *S-*locus supergene, known to be hemizygous in S-morphs and absent from L-morphs in *Primula*, *Fagopyrum Turnera*, and *Linum*^12,17,19,20,24^.

One of the most popular ornamental plants in Europe^27^, *Primula* (primrose) has served as the canonical model to study distyly since Darwin^2^. Extensive genomic resources are available for this genus, including a chromosome-scale genome assembly for *Primula veris*, in which the *S-*locus is a ~ 260 kb region containing five genes (*CCM*^*T*^, *GLO*^*T*^, *CYP*^*T*^, *PUM*^*T*^, *KFB*^*T*^)^12,17^. Two *S-*locus genes have been functionally characterized in *Primula*: *GLO*^*T*^, homologous to the highly-conserved B-class floral homeotic gene *GLOBOSA*, determines high anther position in S-morphs^15^ and *CYP*^*T*^, a member of the cytochrome P450 CYP734A family that degrades brassinosteroids^28^, determines short styles^13^ and female incompatibility in S-morphs^16^. The functions of the other three *S-*locus genes (*CCM*^*T*^, *PUM*^*T*^, *KFB*^*T*^) remain unknown. Four *S-*locus genes originated via gene duplication, and their closest paralogs have been identified (*CCM1*, *GLO1*, *CYP734A51*, *KFB1*)^12,13,17^. A key open question on the evolution of distyly is how the *S-*locus genes acquired their role in controlling distyly. This might have occurred through a change in protein function, temporal and/or spatial gene expression, or a combination thereof, of the *S-*locus genes compared to their respective paralogs^15^. The lack of comparative expression profiles of the *S-*locus genes and their paralogs has so far precluded our understanding of how *S-*locus genes acquired their new functions.

With more than 1.8 million tons produced per year, buckwheat (*Fagopyrum esculentum)* is the most agriculturally important distylous species (www.fao.org/faostat/en/#data/QCL). Thus, knowledge on the genetic control of distyly for this species is of general interest, as it could help improve breeding strategies and artificial selection^29^. Despite its economic importance, little is known on the genetic underpinnings of distyly in *F. esculentum*, except that the *S-*locus is approximately 5.4 Mb long and contains 32 genes^19^, among which is *S-ELF3*, likely to control style length^18^.

*Turnera* is another genus whose distylous species have been studied for more than a century^30^. In recent years, three *S-*locus genes (hemizygous in S-morphs and absent in L-morphs) have been identified: TsSPH1, likely involved in filament elongation; *TsYUC6* (a member of the YUCCA gene family), involved in auxin biosynthesis and likely controlling pollen development; and *TsBAHD*, inactivating brassinosteroids and controlling both female incompatibility and style length, similarly to *CYP*^*T*^ in *Primula*^20,21,31^. Differentially-expressed genes (DEGs) between L- and S-morphs of *Turnera subulata* flowers have been characterized in a recent study^22^. Among these DEGs were several genes related to the phytochrome interacting factor (PIF) signaling network, a large and highly interconnected network that mediates several plant morphogenetics processes, whose key regulators are the PIF transcription factors^32^. This observation, together with the fact that phytochrome-associated pathways can modulate the morphology of sexual organs in *Brassica rapa* when exposed to different red:far-red ratios^33^, led the authors to propose that the recruitment of genes from the PIF network might represent a commonality among *S-*loci controlling distyly in different species^22^. However, this hypothesis has never been tested.

Our study is aimed at better linking the genotypic underpinnings of distyly to its phenotypic expression by investigating the transcriptomes of three distylous species: *P. veris*, *F. esculentum* and *T. subulata*. First, we generated the *P. veris* transcriptome atlas*,* which allowed us to identify a) the expression patterns of *S-*locus genes and their paralogs and b) genes that show constitutive expression across samples, tissues, and developmental stages. Second, we identified genes that are differentially expressed between L- and S-morph flowers, thus likely regulated by the *S-*locus, in *P. veris* and *F. esculentum*. Third, a comparative transcriptomic analysis among *P. veris*, *F. esculentum* and *T. subulata* identified, for the first time, homologous genes that are differentially expressed between L- and S-morphs, thus potentially involved in controlling distyly, in all three species. Last, we tested the previously proposed hypothesis that *S-*loci evolved by recruiting genes from the phytochrome-interacting factor (PIF) signaling network^22^. The results presented here address fundamental questions on the function and evolution of distyly by identifying genes involved in the expression of distyly in *Primula* and *F. esculentum* and revealing commonalities that might explain the convergent evolution of distyly in these two species and *T. subulata*.

## Results and discussion

### *Primulaveris* transcriptome atlas

**Figure 1:**
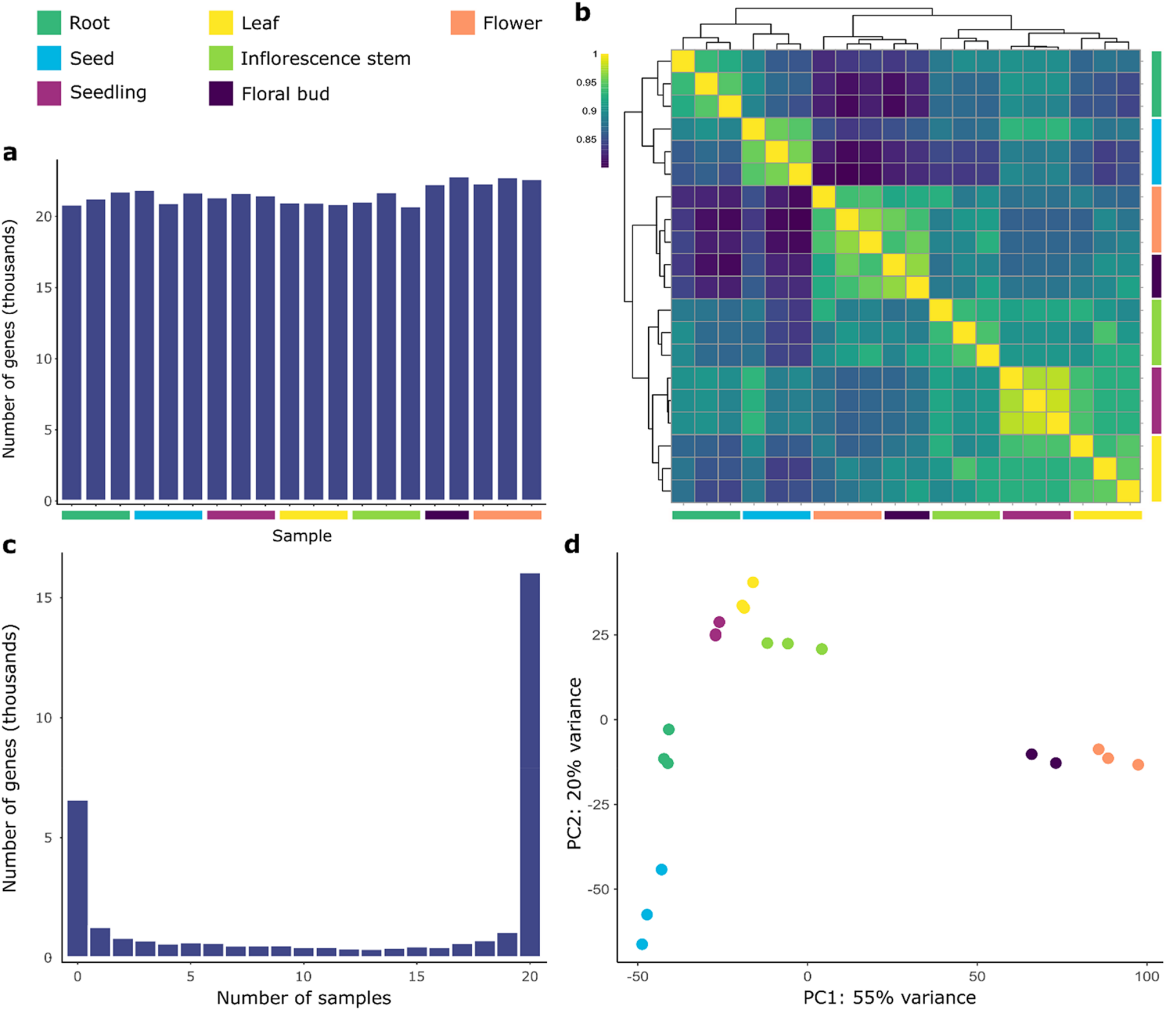
Overview of *P. veris* transcriptomic data. Samples of the same tissue are color coded, following the color scheme in the top-left corner of the figure. (**a**) Number of transcriptionally active genes (i.e. genes with normalized counts ≥ 4) per sample. (**b**) Heatmap of hierarchical clustering of the samples based on their pairwise similarity (estimated as Spearman’s rank correlations) for all 20 samples, with color scale indicating the degree of correlation. (**c**) Number of genes expressed in any given number of samples. (**d**) PCA plot for the 20 RNA-seq samples. Samples belonging to the same tissue cluster together. The first principal component (x-axis) separates samples of reproductive tissues (floral bud (purple) and flower (salmon)) from all remaining tissues.

Twenty RNA-seq samples were used to analyze the transcriptomes of seven tissues (root, seed, seedling, leaf, inflorescence stem, floral bud, and flower) of *P. veris*. A total of 2.27 billion paired-end reads (342.77 Gb; Suppl. Table S1) were used to quantify gene expression of the 34,441 *P. veris* genes (Suppl. Table S2)^17^. Plotting the normalized RNA-seq counts for the genes in each tissue showed, as expected, a bimodal distribution, in which only the peak at higher expression levels comprises the active, functional transcriptome^34^ (Suppl. Fig. S1). Using this distribution and a previously-developed method^35^, we classified a gene as expressed in a tissue if the average number of normalized RNA-seq reads for the replicates of that tissue was ≥ 4. A total of 26,338 genes (76.47%) were expressed in at least one tissue (Fig. 1c). Transcriptional activity did not vary much among tissues, with the number of expressed genes ranging from 21,731 (63.1%) in the seedling to 23,348 (67.8%) in the flower (Fig. 1a). A total of 18,441 genes (53.5%) were expressed across all tissues (Suppl. Fig. S2).

**Figure 2:**
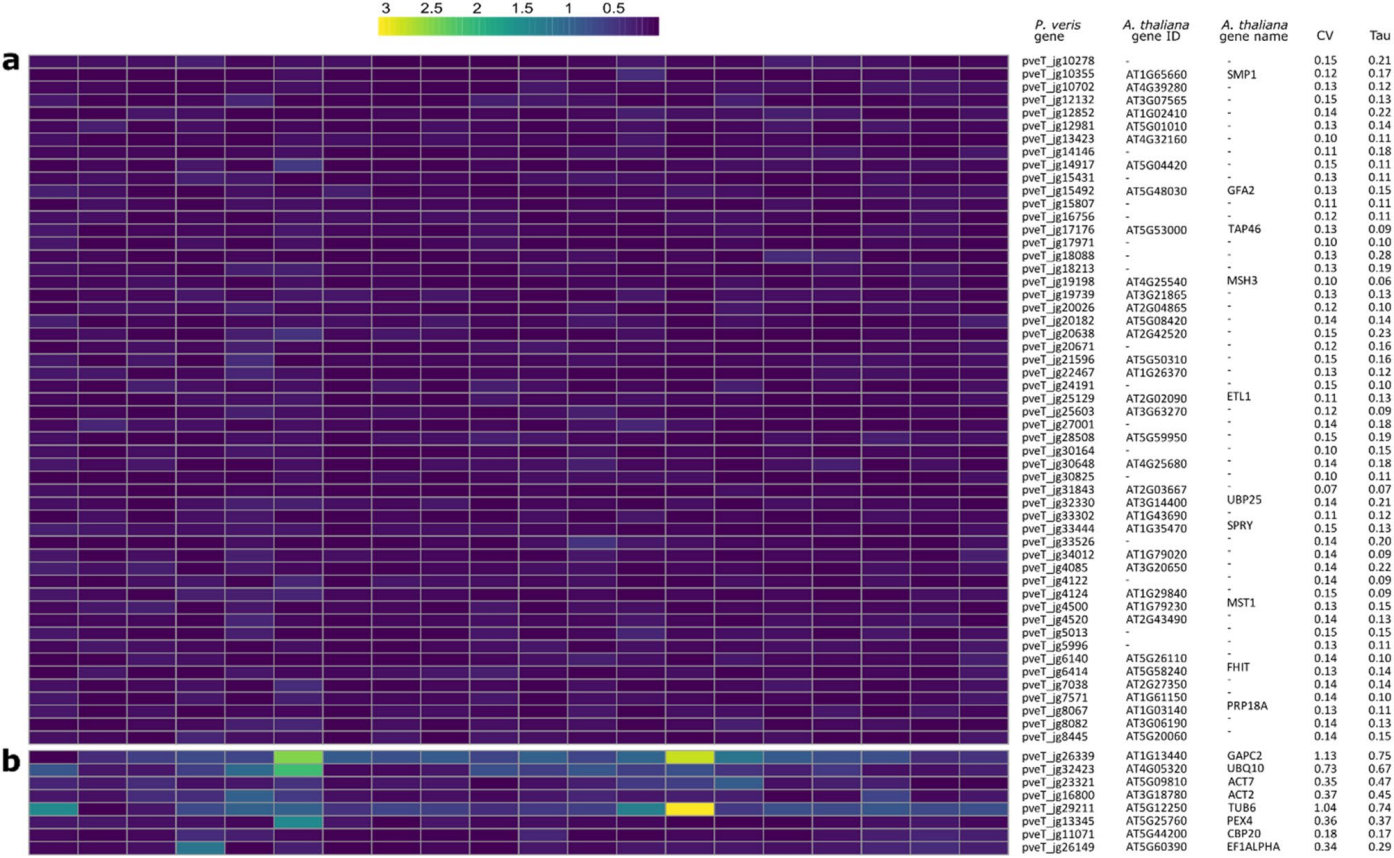
Constitutively expressed genes in *P. veris*. Heatmap showing the variability of expression for the 53 constitutively expressed genes of *P. veris* identified in the current study (**a**) and for eight HKGs commonly used as standards for normalizing qPCR experiments (**b**). Each cell in the heatmap represents the deviation from the mean expression (expressed in normalized read counts) for a gene (row) in a sample (column), normalized by the mean expression for that gene, calculated across all samples. The table on the right reports the *P. veris* gene IDs, the *A. thaliana* ortholog IDs and gene name (when available), the coefficient of variation (CV) and the tau tissue-specificity index.

RNA-seq data have proven to be important for the identification of constitutively expressed genes, i.e. genes whose expression does not considerably vary across tissues, developmental stages, environmental conditions and experimental factors^36^. Constitutively expressed genes are especially important in real-time qPCR experiments, where they are used as internal controls for normalizing gene expression among samples^36^. Here, 53 constitutively expressed genes, defined as having a coefficient of variation (CV = standard deviation/mean of normalized counts) ≤ 0.15, were identified (Fig. 2a). The degree of tissue specificity was calculated for each gene using the tau (τ) index^37^ (Suppl Fig S3): the low (< 0.3) tau values found in the constitutively expressed genes further supported their constitutive expression across tissues (Fig. 2a). Housekeeping genes (HKGs) are often used as normalizing factors in qPCR experiments, but such an approach can introduce bias in the quantification of gene expression, as many HKGs have been shown not to be constitutively expressed across tissues and developmental stages^38^. We noticed that none of the eight HKGs commonly used as reference in qPCR experiments^36^ was found to be constitutively expressed in *P. veris* (all having CV > 0.15), demonstrating once again the importance of identifying species-specific constitutively expressed genes through RNA-seq^36^ (Fig. 2b). The set of *P. veris* constitutively expressed genes identified here represents a useful resource for normalizing gene expression in *Primula* qPCR analyses.

The expression patterns of the 20 *P. veris* samples matched the biological nature of the respective tissues, as shown by their hierarchical clustering (Fig. 1b). The highest Spearman's rank correlation coefficients were found between samples of the same tissue; between tissues, floral bud and flower samples shared the highest Spearman's coefficients (ρ = 0.95), as expected since they represent two developmental stages of the same organ (Suppl. Fig. S4). Samples from the same tissue clustered together also in a PCA plot, which additionally showed a clear distinction between samples of floral (floral buds, flowers) and non-floral tissues (roots, seeds, seedlings, leaves, and inflorescence stems) on the first principal component (Fig. 1d).

### Expression profiles of S-locus genes and their paralogs in *P. veris*

With the exception of *CYP*^*T*^ and *GLO*^*T*^, known to be expressed exclusively in style and corolla tube, respectively^13,15^, the expression profiles of the *P. veris S-*locus genes were previously unknown. Given this lack of knowledge, elucidating how the *S-*locus genes acquired their role in controlling distyly after their origin via duplication has so far been impossible. To better characterize the expression profiles of all *S-*locus genes and their paralogs, we performed differential gene expression analyses between samples of floral and non-floral tissues. This allowed us to test if the duplicate *S-*locus genes diversified their expression profiles compared to their respective paralogs, which might have resulted in them gaining their functions in controlling distyly.

**Figure 3:**
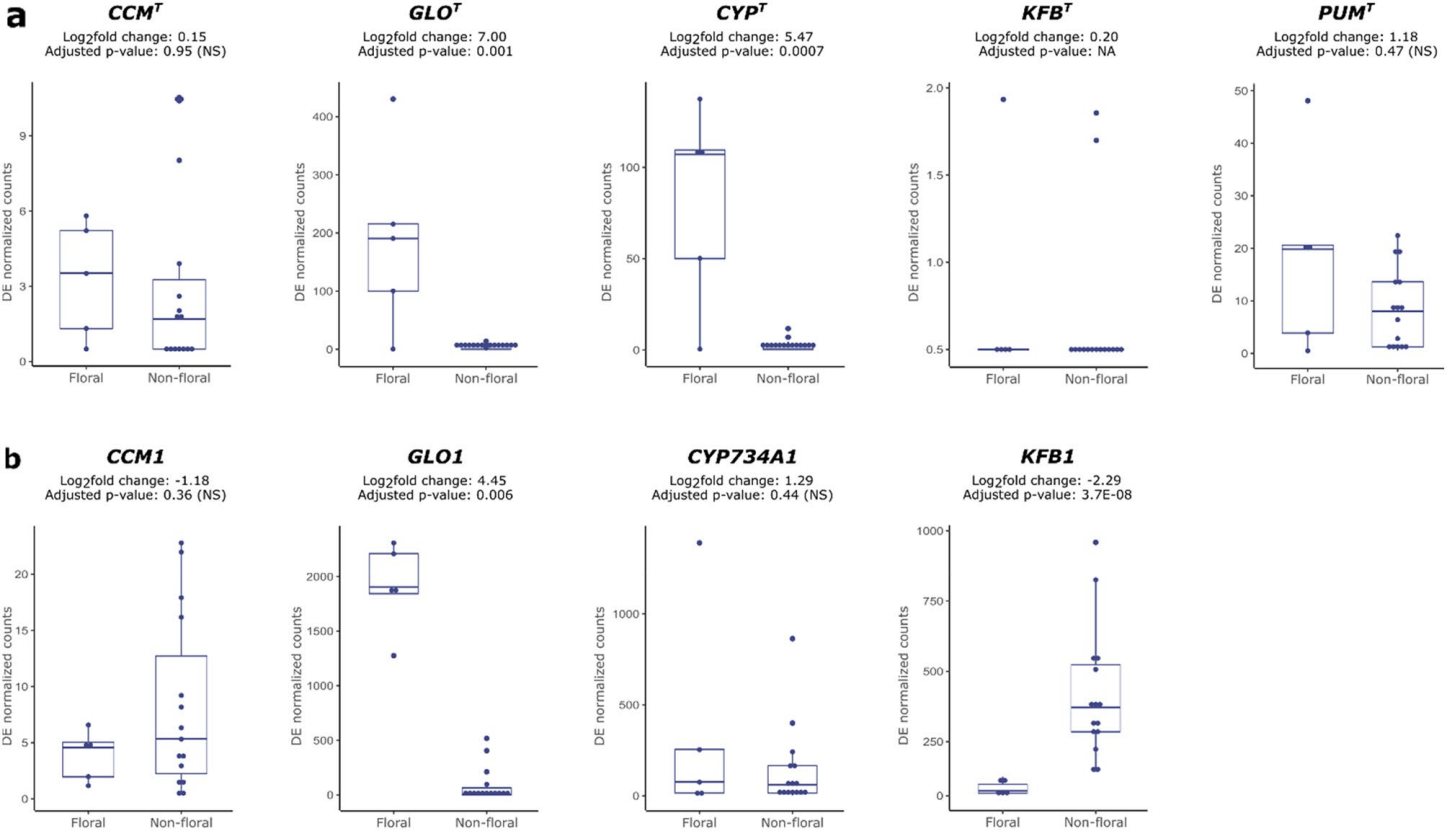
Expression profile of S-locus genes and their paralogs in *P. veris*. Box-plots showing the expression (as normalized counts) of S-locus genes (**a**) and their paralogs (**b**) in samples of floral (floral bud and flower; n = 5) and non-floral (root, seed, seedling, leaf, inflorescence stem; n = 15) tissues. For each gene, the log2fold change of expression between floral and non-floral tissues is reported together with the adjusted *p* value, calculated by DESeq2. NA, not applicable; NS, not significant.

Of the five *S-*locus genes, *GLO*^*T*^ and *CYP*^*T*^ were significantly more expressed in floral than non-floral tissues, confirming previous results^13,15^ (adjusted *p* value < 0.01); *PUM*^*T*^ and *CCM*^*T*^ were expressed at roughly the same level in floral and non-floral tissues; *KFB*^*T*^ appeared not to be expressed in any tissue (normalized counts < 4) (Fig. 3a). Since *KFB*^*T*^ was reported to be expressed in *P. vulgaris* flower (see ref.^12^ and below), we believe that we did not detect *KFB*^*T*^ expression in *P. veris* because it was not expressed at the time when the floral tissues were harvested, rather than not being expressed at all. This observation may also be indicative of *KFB*^*T*^ being expressed only for a short temporal window. Furthermore, the lack of flower-specific expression for *CCM*^*T*^ and *PUM*^*T*^, together with the fact that their functions are still unknown, leaves open the question of whether these genes play a role in distyly. Functional studies on *CCM*^*T*^ and *PUM*^*T*^ will be necessary to address this question.

Among the four *S-*locus gene paralogs, only *GLO1* was significantly more expressed in floral than non-floral tissues (as expected, as it is a B-class floral homeotic gene^15^), while no significant differences between tissue types were found for *CCM1* and *CYP734A51*, and *KFB1* was significantly more expressed in non-floral than floral tissues (Fig. 3b). Thus, the flower-confined expression of *CYP*^*T*^ is not shared with its closest paralog (*CYP734A51*). This suggests that *CYP*^*T*^ acquired its role in controlling distyly via a change in the expression profile compared to its paralog; whether this was accompanied by a change in its protein function remains to be tested. This observation marks a difference on how the two *S-*locus genes with a known function (*GLO*^*T*^ and *CYP*^*T*^) acquired their involvement in controlling distyly, i.e. *GLO*^*T*^ through mutations that changed the activity of its encoded protein^15^ but no changes in its expression profile, while *CYP*^*T*^ through changes in its expression profile that limited its expression to the style but potentially no changes in its protein function.

### Differential expression analysis between L- and S-morph flowers

To better link the genotypic underpinnings of heterostyly to its phenotypic expression, we identified differentially expressed genes (DEGs) between L- and S-morph flowers using RNA-seq data from different samples of *P. veris* (style and corolla tube), *P. vulgaris* (whole flower) (Suppl. Table S3), and *F. esculentum* (stamen filament and corolla tube; Suppl. Table S4). As the *S-*locus is the only region consistently differing between L- and S-morph genomes, we hereinafter refer to any gene up-regulated in S- compared to L-morphs as ‘up-regulated’ and any gene down-regulated in S- compared to L-morphs as ‘down-regulated’, implying that these genes are up- or down-regulated by the *S-*locus.

#### Differential expression analysis in *Primula*

**Figure 4:**
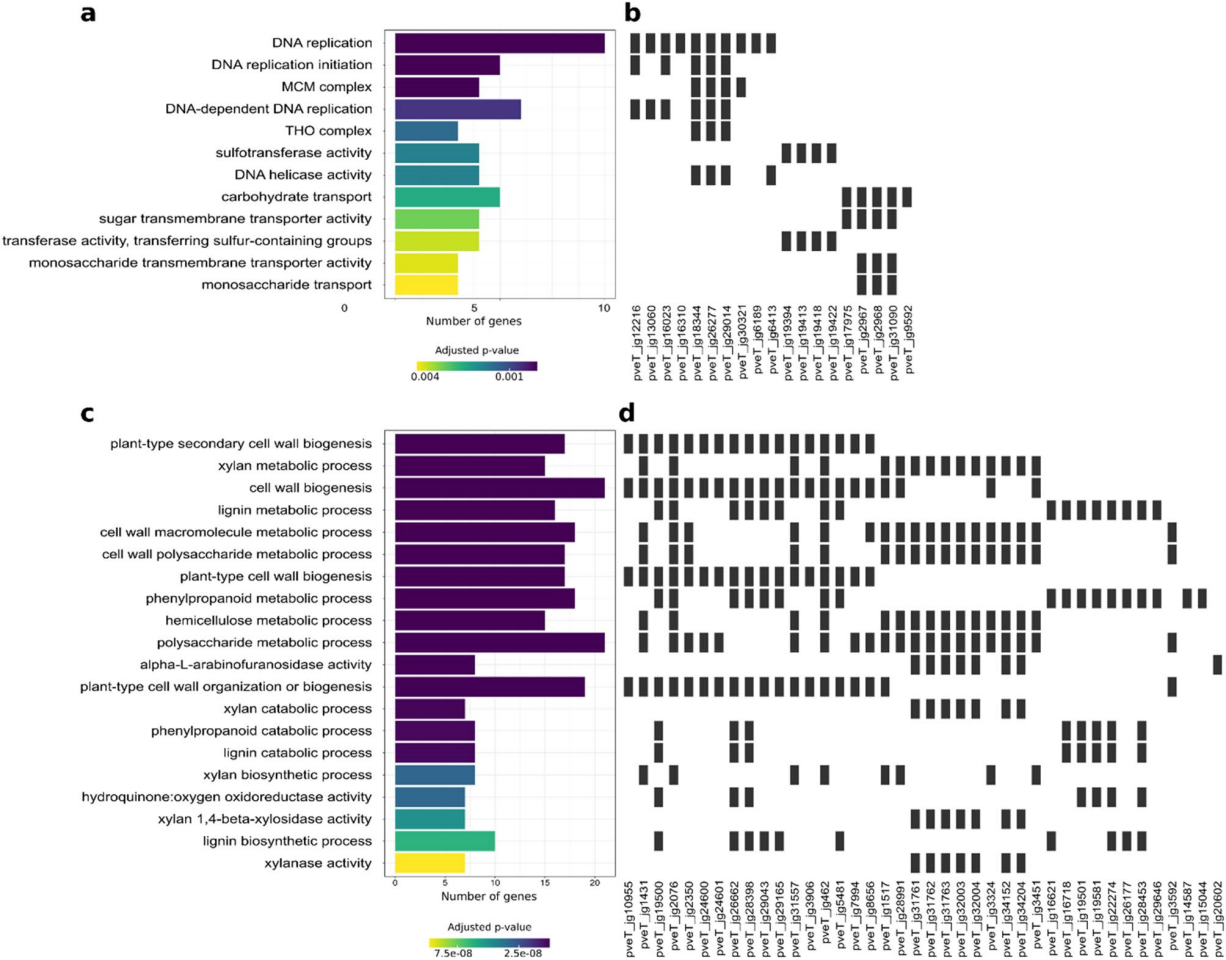
Differential expression analysis in *Primula*. (**a**) Bar-plot showing the enriched GO terms among down-regulated DEGs in the *P. veris* style, ordered bottom-up by increasing adjusted *p* values. The bar length is proportional to the number of genes belonging to each GO category (x-axis). (**b**) Heat-plot showing which genes (x-axis) belong to which GO category. (**c**), (**d**) Same as a and b, but for the 20 most significantly enriched GO terms among up-regulated DEGs in the *P. vulgaris* flower (full list in Suppl. Table S10).

In the *P. veris* style, 245 DEGs (78 up- and 167 down-regulated) were identified (Suppl. Table S5). Of the *S-*locus genes, *GLO*^*T*^, *CYP*^*T*^ and *PUM*^*T*^ (but not *CCM*^*T*^ and *KFB*^*T*^) were found in the up-regulated gene set. Eight DEGs were involved in cell wall modifications, among which was the up-regulated pveT_jg12120, homologous to *A. thaliana* IBL1, a IBH1-like transcription factor known to negatively regulate cell elongation in response to brassinosteroid signaling^39^. A GO analysis on the down-regulated genes revealed an enrichment of terms related to two main categories: DNA replication and sugar transport (Fig. 4a,b; Suppl. Table S6). Among the genes associated to “DNA replication” (GO:0006260), “DNA replication initiation” (GO:0006270) and “DNA-dependent DNA replication” (GO:0006261) were key regulators of the cell cycle, such as: pveT_jg12216, homologous to *A. thaliana* Cell Division Cycle 6 (*CDC6*; AT1G07270), which confers cells the ability to initiate DNA replication^40^; pveT_jg13060, homologous to an *A. thaliana* transcription factor (AT3G02820), member of the zinc knuckle (CCHC-type) protein family, which is required for cell cycle progression and DNA replication upon regulation by E2F transcription factors^41^; pveT_jg18344, pveT_jg26277, pveT_jg29014, pveT_jg30321, and pveT_jg16023, all putative members of the Minichromosome Maintenance (MCM) protein family, which is pivotal in the initiation of DNA replication^42^. GO terms related to sugar transport were “carbohydrate transport” (GO:0008643), “sugar transmembrane transporter activity” (GO:0051119), and “monosaccharide transport” (GO:0015749) and comprised, among others, pveT_jg31090, homologous to *A. thaliana* Sugar Transporter 1 (*STP1*; AT1G11260).

Of the 143 DEGs identified in the *P. veris* corolla tube, 74 were up-regulated, which included all *S-*locus genes except *KFB*^*T*^, and 69 down-regulated (Suppl. Table S7). A GO enrichment analysis on this gene set did not provide useful information, as the enriched GO terms were not related to developmental processes that could be associated to the floral dimorphism (Suppl. Table S8). Among the up-regulated genes was pveT_jg5930, homologous to a WALLS ARE THIN1 (WAT1)-related protein. *WAT1* encodes for an auxin transporter which regulates secondary cell wall thickness and auxin transport in *A. thaliana*^43^.

In the *P. vulgaris* whole flower, 268 DEGs were identified, 198 being up- and 70 down-regulated (Suppl. Table S9). The up-regulated gene set, which contained all *S-*locus genes except *CCM*^*T*^, showed an enrichment in GO terms related to cell-wall modifications, such as “plant-type secondary cell wall biogenesis” (GO:0009834), “polysaccharide metabolic process” (GO:0044264), and “cell wall biogenesis” (GO:0042546) (Fig. 4c,d; Suppl. Table S10). The down-regulated gene set showed an enrichment in genes associated to “carbohydrates transport” (GO:0008643), driven by three genes annotated as members of the TC 2.A.1.1 sugar transporter family (pveT_jg2967, pveT_jg2968 ,pveT_jg2971) and by a UDP-galactose UDP-glucose transporter (pveT_jg9592).

Research on the mechanisms underlying style length dimorphism in *Primula* has shown a predominant role of cell elongation. The *S-*locus gene *CYP*^*T*^, which is expressed exclusively in the style, degrades brassinosteroids, thus limiting cell elongation and ultimately resulting in a shorter style in S-morphs than in L-morphs^5,13^. However, it has previously been proposed that differential cell division might also affect differential style length between morphs^8^, following the observation that L-morph styles are usually twice the length of S-morph styles but L-morph style cells are not twice as long as S-morph style cells^5^. The down-regulation of genes involved in DNA replication observed in the S-morph style suggests that this tissue is undergoing reduced cell division, thus supporting the hypothesis that cell division also contributes to style-length dimorphism. Furthermore, the decrease in sugar transport may also be responsible for the style-length dimorphism, as carbohydrates play a key role in both cell expansion and division^44^.

#### Differential expression analysis in *F. esculentum*

**Figure 5:**
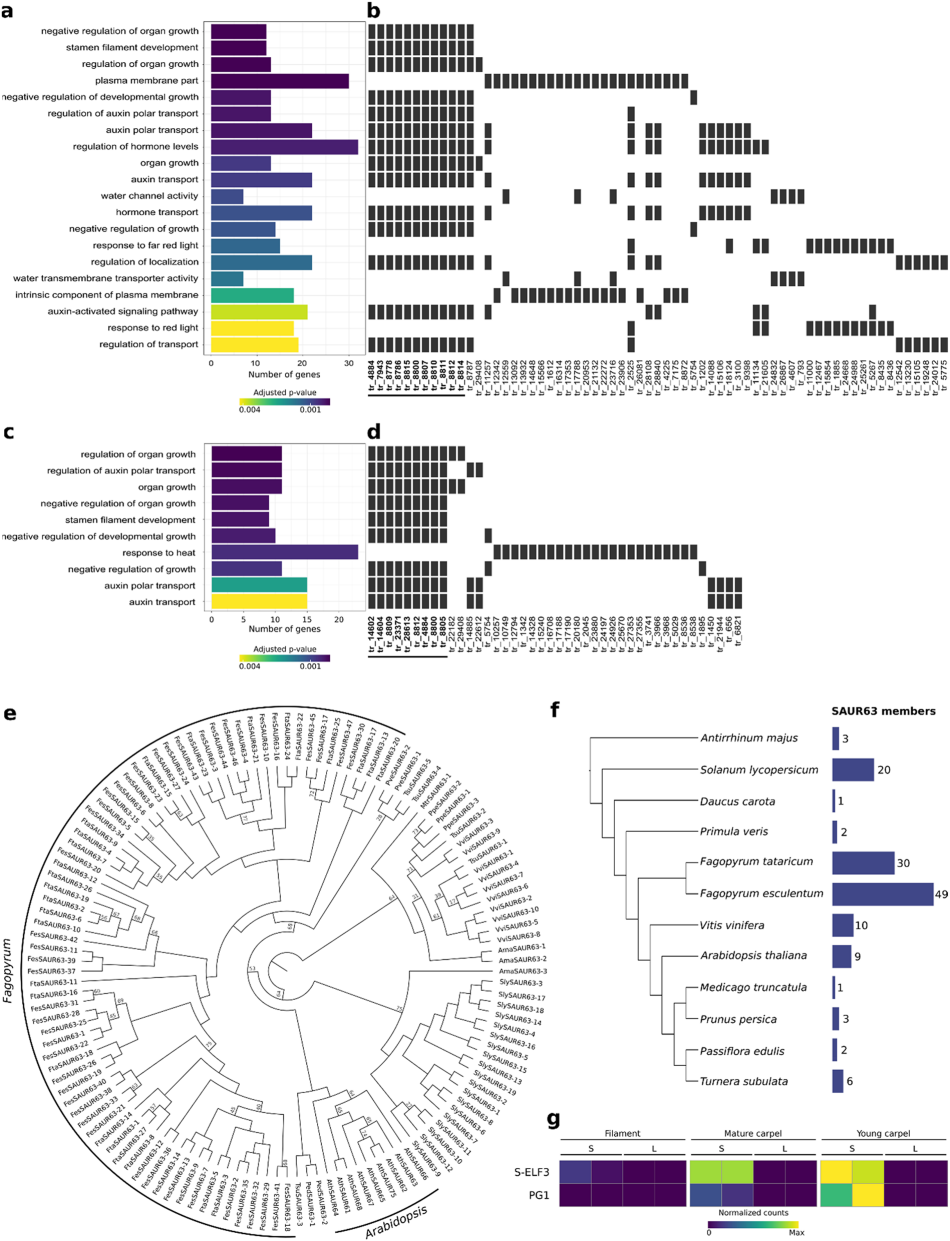
Differential expression analysis in *F. esculentum*. (**a**) Bar-plot showing the 20 most significantly enriched GO terms among down-regulated DEGs in the stamen filament, ordered bottom-up by increasing adjusted *p* values. The bar length is proportional to the number of genes belonging to each GO category. (**b**) Heat-plot showing which genes (x-axis) belong to which GO category. To ease visualization, genes associated to only one GO term were removed (full list in Suppl. Table S12). Genes belonging to the OG0000060 orthogroup (i.e. putatively coding for SAUR63 proteins) are boldfaced and highlighted by a black horizontal bar. (**c**), (**d**) Same as a and b, but for all significantly enriched GO terms among down-regulated genes in the mature carpel. (**e**) Phylogeny of the OG0000060 orthogroup. Bootstrap values < 75 are shown. To ease visualization, genes from *A. majus*, *S. lycopersicum*, *P. veris*, *F. tataricum*, *F. esculentum*, *V. vinifera*, *A. thaliana*, *M. truncatula*, *P. persica*, *P. edulis*, and *T. subulata* are indicated with the prefixes Ama, Sly, Pve, Fta, Fes, Vvi, Ath, Mtr, Ppe, Ped, Tsu, respectively (details on the nomenclature in Suppl. Table S18). (**f**) Cladogram of the twelve angiosperm species (modified from Janssens et al. 2020) used in our OrthoFinder analysis with the number of putative SAUR63 subfamily members identified in each species (i.e. the number of genes in the OG0000060 orthogroup). (**g**) Heatmap showing the normalized counts in three tissues (filament, mature carpel and young carpel) of two floral morph (S and L) for the S-locus genes S-ELF3 and PG1. For plotting, the normalization was performed for each gene independently. Maximum values correspond to 311.9 and 2513.5 normalized counts for S-ELF3 and PG1, respectively; minimum value is zero for both genes.

Two *S-*locus genes have so far been identified in *F. esculentum*, namely *S-ELF3* and *SSG2*^18,19^, while a third gene (*PG1*) has been shown to be expressed exclusively in S-morph styles, despite not being physically linked to the *S-*locus^45^. We identified both *S-ELF3* and *PG1* (but not *SSG2*) in the *F. esculentum* gene set^46^, as tr_15748 and tr_3984, respectively (see Methods). *PG1* was expressed only in S-morph carpel samples, while *S-ELF3* was expressed also in the filament, albeit at lower level (Fig. 5g).

In the filament, 1316 genes were differentially expressed between the two morphs (553 up- and 763 down-regulated; Suppl. Table S11). A GO enrichment analysis revealed a set of 12 down-regulated genes (tr_4884, tr_7943, tr_8778, tr_8786, tr_8787, tr_8800, tr_8807, tr_8810, tr_8811, tr_8812, tr_8814, tr_8815) associated to GO terms that indicate a negative role of these genes in stamen filament development, mediated by auxin: “negative regulation of organ growth” (GO:0046621), “auxin transport” (GO:0060918), and “stamen filament development” (GO:0080086) (Fig. 5a,b; Suppl. Table S12).

In the young carpel, 825 DEGs (299 up- and 526 down-regulated) were identified (Suppl. Table S13). The GO categories “pectinesterase activity” (GO:0030599) and “cell wall organization” (GO:0071555) were significantly enriched among down-regulated genes (Suppl. Table S14). Of the 955 DEGs identified in the mature carpel (402 up- and 553 down-regulated; Suppl. Table S15), a group of eleven down-regulated genes (tr_14602, tr_14604, tr_22182, tr_23371, tr_28613, tr_29408, tr_4884, tr_8800, tr_8805, tr_8809, tr_8812) was associated to GO terms suggesting their role in auxin-mediated organ growth, such as “regulation of organ growth” (GO:0046620) and “auxin transport” (GO:0060918) (Fig. 5c,d; Suppl. Table S16).

Noting the overlap between filament and carpel down-regulated genes, we further investigated these genes by identifying their homologous genes in eleven additional angiosperms via an OrthoFinder analysis. All the auxin- and growth-related genes that were down-regulated in the filament (except tr_8787) and in the mature carpel (except tr_22182 and tr_29408) were in the OG0000060 orthogroup, which also contained the *A. thaliana SMALL AUXIN UP RNA* (*SAUR*)*63* subfamily (i.e. *SAUR61-68* and *SAUR75*) members AT1G29420, AT1G29430, AT1G29440, AT1G29450, AT1G29460, AT1G29500, AT1G29510, AT1G29490, and AT5G27780. Members of the *SAUR63* subfamily are known to play a role in hypocotyl and stamen filament elongation by activating plasma membrane H^+^-ATPases in response to auxin, thus promoting cell elongation^47,48^. We found that the two *Fagopyrum* species (*F. esculentum* and *F. tataricum*) displayed the highest number of *SAUR63* members (49 and 30, respectively; Fig. 5f) among all the angiosperms included in our gene orthology analysis. To better characterize the relationships among *SAURs*, we generated a phylogeny for the OG0000060 orthogroup, which showed that the *SAUR63* expansion observed in *Fagopyrum* is independent from the one observed in *Arabidopsis* (Fig. 5e).

The results above imply that auxin is likely the main hormone controlling the differential elongation of stamen and pistil in *F. esculentum*, potentially favored by a *Fagopyrum*-specific expansion of the *SAUR63* subfamily, whereas brassinosteroids were shown to be the main mediator of differential style elongation in *Primula*^13^.

### Comparative transcriptomics among distylous species

**Figure 6:**
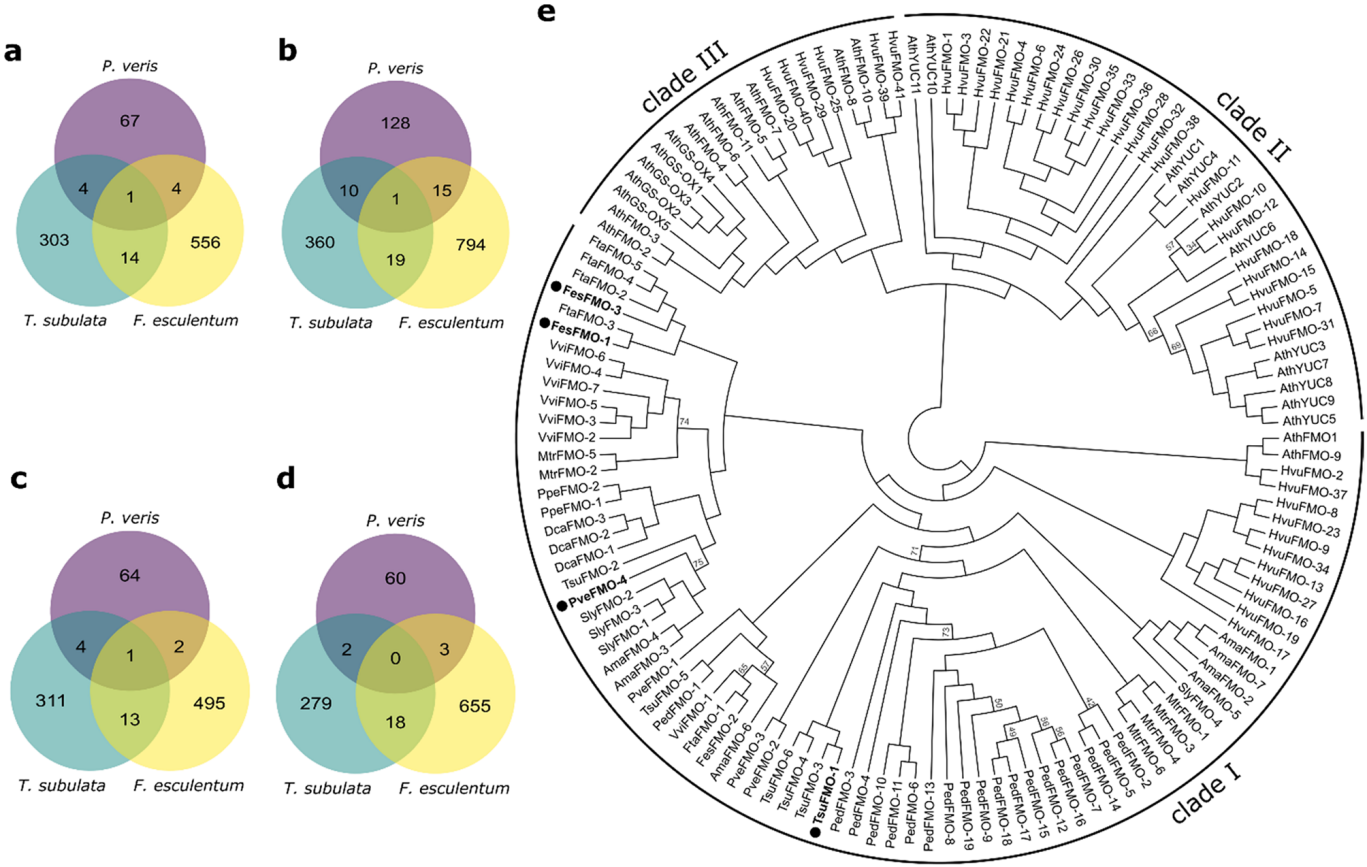
Orthogroups containing genes differentially expressed in *P. veris*, *F. esculentum* and *T. subulata*. (**a**)–(**d**) Venn diagrams showing the number of orthogroups containing differentially expressed genes between L- and S-morphs of *P. veris* (purple), *F. esculentum* (yellow) and *T. subulata* (green). (**a**) Orthogroups containing genes up-regulated in female organs. One orthogroup (OG0000318) contains genes up-regulated in female organs in the three species (pveT_jg29738, tr_14668, Tsub_00016967-RA), annotated as HSL3 receptor kinases. (**b**) Orthogroups containing genes down-regulated in female organs. One orthogroup (OG0000198) contains genes down-regulated in female organs in the three species (pveT_jg28601, tr_16287, Tsub_00008119-RA, and Tsub_00000118-RA), annotated as PIP aquaporins. (**c**) Orthogroups containing genes up-regulated in male organs. One orthogroup (OG0000315) contains genes up-regulated in male organs in the three species (pveT_jg17457, tr_6752, Tsub_00026904-RA, and Tsub_00006450-RA). (**d**) Orthogroups containing genes down-regulated in male organs. No orthogroup contains genes down-regulated in male organs in the three species. (**e**) Cladogram of the OG0000315 orthogroup genes, which contains genes annotated as FMOs, plus *A. thaliana* and *H. vulgare* FMOs. Bootstrap values < 75 are shown. The *P. veris*, *F. esculentum* and *T. subulata* DEGs are boldfaced. To ease visualization, genes from the different species are indicated with the prefixes as in Fig. 5f, plus *H. vulgare*, and *D. carota*, indicated as Hvu and Dca, respectively (details on the nomenclature in Suppl. Table S19. Genes that were differentially expressed between L- and S-morphs are boldfaced and marked by black circles.

Distyly evolved independently at least 13 times^9^, representing a classic example of evolutionary convergence^10^. In the present study we identified DEGs between L- and S-morph pistils and stamens of *P. veris* and *F. esculentum*. The availability of DEGs between L- and S-morph flowers of *Turnera subulata*^22^ (Table 1) allowed us to perform a three-way comparative transcriptomics analysis aimed at discovering whether some genes are potentially involved in the control of distyly in all three species. Since it is difficult to identify one-to-one orthologs among distantly related species, we performed an OrthoFinder analysis including the proteomes of 12 angiosperms (see Methods for details) and searched for any orthogroup containing genes that were differentially expressed in all three species (Suppl. Table S17). This search was performed separately for up-regulated genes in female organs (Fig. 6a), down-regulated genes in female organs (Fig. 6b), up-regulated genes in male organs (Fig. 6c), and down-regulated genes in male organs (Fig. 6d). Of these, only the set of genes down-regulated in male organs did not have any shared orthogroup (Fig. 6d).

**Table 1:** Summary of differentially expressed genes (DEGs) in distylous species.

Species	Tissue	DEGs			Reference
		Total	Up-regulated	Down-regulated	
*Primula veris*	Style	245	78	167	Present study
	Corolla tube	143	74	69	
*Primula vulgaris*	Whole flower	268	198	70	
*Fagopyrum esculentum*	Mature carpel	955	402	553	
	Young carpel	825	299	526	
	Stamen filament	1,316	553	763	
*Turnera subulata*	Young pistil	253	111	142	Henning et al.^22^
	Young stamen	369	224	145	
	Mature pistil	537	246	291	
	Mature stamen	338	147	191

One orthogroup (OG0000315) was found to contain four genes that were up-regulated in the male organs of the three species (pveT_jg17457, tr_6752, Tsub_00026904-RA, and Tsub_00006450-RA; Fig. 6c). All OG0000315 genes were functionally annotated as flavin-containing monooxygenases (FMOs). FMOs belong to a large and highly-conserved protein family whose function is to incorporate an oxygen atom from molecular oxygen into small nucleophilic or electrophilic molecules^49^. This finding is of particular interest for two reasons. First, FMOs overlap in function with cytochrome P450 monooxygenases, a superfamily of enzymes that also includes the protein encoded by *CYP*^*T*^, the *S-*locus gene that controls style length and female incompatibility in *P. veris*^13,16^. Second, the *T. subulata S-*locus gene *YUC6*, which controls male mating type and pollen size^23^, is a FMO, specifically a member of the *YUCCA* gene family which catalyzes the second step in auxin synthesis from L- tryptophan^20,50^. To better characterize the putative function of these differentially expressed FMOs, we generated a phylogeny that included the sequences of OG0000315 genes as well as those of *A. thaliana* and barley (*Hordeum vulgare*) FMOs^51^ (Fig. 6e). The OG0000315 genes fall in the FMO Clade I, whose genes are supposedly involved in pathogen defense^52^, and to our knowledge are not involved in floral development in any species. Whether pveT_jg17457, tr_6752, Tsub_00026904-RA, and Tsub_00006450-RA play a role in controlling distyly thus remains unclear, and functional investigations would be required to clarify this point.

Another orthogroup (OG0000318) included three genes up-regulated in the female organs of the three species (pveT_jg29738, tr_14668, Tsub_00016967-RA; Fig. 6a), all annotated as members of the Serine/Threonine protein kinases family. Specifically, these genes are homologous to *A. thaliana HAESA-LIKE 3* (*HSL3*; AT5G25930), a highly-conserved leucine-rich repeat receptor kinase (LRR-RK) which forms a complex with *BRASSINOSTEROID INSENSITIVE 1-ASSOCIATED KINASE 1* (*BAK1*) upon induction by a class of small signaling peptides named CTNIPs^53^. The CTNIP-HSL3 signaling pathway seems to be involved in stress response but also affects plant growth, although it remains unknown whether it plays a role in floral development ^53^. This observation, together with the fact that *BAK1* plays a pivotal role in regulating brassinosteroid-dependent growth^54,55^, suggests the involvement of brassinosteroids in causing the style length dimorphism not only in *Primula* and *T. subulata*^13,21^ but also in *F. esculentum*.

Finally, a third orthogroup (OG0000198) contained genes down-regulated in the female organs of the three species (pveT_jg28601, tr_16287, Tsub_00008119-RA, and Tsub_00000118-RA; Fig. 6b). All OG0000198 genes were annotated as aquaporins and, more specifically, the eight *A. thaliana* genes in this orthogroup were annotated as plasma membrane intrinsic proteins (PIPs). Aquaporins are integral membrane proteins that function as water channels but also mediate the transport of other important substrates and play a key role in plant growth and development by regulating cell turgor, thus cell expansion^56–58^. Indeed, the expression of PIP aquaporins has been shown to strongly correlate with cell expansion and tissue growth in several plant species^59–61^. Thus, the decreased expression of PIP aquaporins in S-morph pistils is compatible with the shortening of the style in this floral morph, due to reduced cell expansion.

Our results show that, even though the three species tested here are distantly related and evolved distyly independently^9^, some genes are differentially expressed in the same organs between L- and S-morph flowers, hence some shared biological pathways might underlie the expression of distyly in these three species. Thus, the convergent evolution of distyly at the phenotypic level is mirrored by some convergence also at the molecular level, representing one of the first studied cases of convergent evolution of complex traits^62,63^.

### Enrichment analysis of PIF-regulated genes among DEGs

We tested the previously proposed hypothesis that the recruitment of genes intersecting with the PIF signaling network is a common motif in the evolution of distyly supergenes^22^ by verifying whether the DEGs identified between L- and S-morphs of *Primula*, *F. esculentum* and *T. subulata* were enriched in genes known to be PIF-regulated. If *S-*locus genes are indeed related to PIF network hubs, we expect an enrichment of PIF-regulated genes among DEGs.

Of the ten floral tissues analyzed, six showed a significant enrichment in PIF-regulated genes among DEGs compared to the genome background (Fisher’s exact test; *p* value < 0.05; Table 2). In *T. subulata* we found an enrichment of PIF-regulated genes in female organs (young and mature pistil), but not in male organs (young and mature stamen). In *Primula*, both style and corolla tube (but not whole flower) DEGs were enriched in PIF-regulated genes. In *F. esculentum*, DEGs identified in the mature carpel and in the filament (but not those identified in the young carpel) showed an enrichment in PIF-regulated genes. Of particular interest in this regard was the identification of the SAUR63 subfamily as a putative modulator of stamen filament and pistil elongation in *F. esculentum* (see above), as SAURs can induce organ growth by promoting cell elongation^64^ and can be up-regulated by auxin, brassinosteroids (the two key hormones in the floral dimorphism), and PIFs^64^.

**Table 2:** Enrichment analysis of PIF-regulated genes among DEGs in ten samples.

		DEGs			Non-DEGs			*p* value	
		Total	PIFs	Non-PIFs	Total	PIFs	Non-PIFs		
*Primula veris*	Style	95	12	83	10,775	513	10,262	0.002	●
	Corolla tube	40	6	34	10,832	520	10,312	0.012	●
*Primula vulgaris*	Whole flower	82	3	79	10,785	523	10,262	0.766	
*Fagopyrum esculentum*	Mature carpel	199	25	174	9459	418	9041	< 0.001	●
	Young carpel	144	10	134	9516	434	9082	0.126	
	Stamen filament	381	43	338	9275	401	8874	< 0.001	●
*Turnera subulata*	Mature pistil	214	45	169	8908	417	8491	< 0.001	●
	Mature stamen	108	9	99	9014	453	8561	0.096	
	Young pistil	78	14	64	9044	448	8596	< 0.001	●
	Young stamen	112	9	103	9010	453	8557	0.114	

Black circles at the end of the rows indicate samples in which DEGs are significantly enriched in PIF-regulated genes.

These results demonstrate that genes known to be part of the PIF signaling network are enriched among DEGs between S- and L-morph flowers of three species that evolved distyly independently. Thus, *S-*loci might indeed evolve via the recruitment of PIF-related genes, as previously proposed^22^.

## Conclusions

We generated a transcriptome atlas for the distylous *P. veris*, which allowed us to identify a set of 53 genes that are constitutively expressed across tissues, representing a useful resource for normalizing gene expression in qPCR experiments (Fig. 2). Thanks to extensive transcriptomic data from multiple floral and non-floral tissues of *P. veris*, we could also determine that the *S-*locus gene *CYP*^*T*^ likely acquired its role in distyly via a change in expression profile, compared to its closest paralog (Fig. 3).

A differential gene expression analysis between L- and S-morph flowers confirmed that in *Primula* the differential style elongation between the two morphs is caused by a difference in style cell expansion (up-regulation of genes involved in cell-wall modification in the *P. vulgaris* flower; Fig. 4c,d), but also revealed a potential role of cell division, as implied by the down-regulation of genes associated to DNA replication and sugar transport in the S-morph style (Fig. 4a,b). In *F. esculentum*, a set of 17 SAURs was linked to the differential elongation of both stamen filament and pistil, indicating auxin, rather than brassinosteroids, as the main growth-inducing hormone determining the floral dimorphism in this species (Fig. 5).

In conclusion, this is the first study that identifies the main differences and commonalities in the genetic underpinnings of distyly among distantly related taxa (here, *P. veris*, *F. esculentum* and *T. subulata*; Table 1, Fig. 6). The main difference concerns the hormones involved in the control of style elongation, which appear to be mediated mainly by brassinosteroids in *Primula* and *Turnera*, and auxin in *Fagopyrum*. On the other hand, two main commonalities emerged. First, we identified three groups of homologous genes that were differentially expressed between L- and S-morphs in the three species studied here, all potentially involved in the phenotypic expression of distyly. Second, DEGs identified in the three species mentioned above are enriched in genes intersecting with the PIF signaling network, thus supporting the hypothesis that distyly supergenes evolved via the recruitment of PIF-related genes (Table 2).

This is the first time that specific genes have been identified as shared players in the expression of distyly in distantly related taxa; the increasing availability of genomic resources for distylous species will clarify whether the shared patterns observed in the three species studied here are also shared among all distylous species or not.

## Methods

### Data retrieval

All transcriptomic data used in this study was downloaded from NCBI GenBank. All *Primula* RNA-seq samples comprised paired-end Illumina reads. The RNA-seq data used for generating the *P. veris* transcriptome atlas consisted in 20 samples belonging to seven tissues, originally published in ref.^17^ and available under the BioProject PRJEB44353 (33.5–84.2 M reads per sample). These 20 *P. veris* samples consisted of three replicates per each tissue (except floral buds, which had only two replicates) and floral tissues contained both L- and S-morph individuals pooled together. For *Primula*, differential expression analyses between L- and S-morphs were performed on three tissues: *P. veris* style (three L- and three S-morph samples), *P. veris* corolla tube with attached anthers (three L- and three S-morph samples), and *P. vulgaris* whole flower (four L- and four S-morph samples). *Primula veris* samples are available under BioProject PRJNA317964 (22.2–39.0 M reads per sample) and were prepared as follows^13^: styles and corolla tubes with attached anthers were harvested from 25 plants per sample when petals were 4–10 mm long, i.e. when visible differences in style and anther position first arise^8,13^. *Primula vulgaris* samples are available under BioProject PRJEB9683 (15.6–25.5 M reads per sample) and were prepared from 15 to 20 mm buds, each sample representing a single individual^12^. The *F. esculentum* RNA-seq data consisted of single-end Illumina reads, were originally published in ref.^46^ and available under BioProject PRJNA487842, and generated from three tissues: stamen filament of mature (10-weeks old) flower, carpel of mature (10-weeks old) flower, carpel of young (8-weeks old) flower; two L- and two S-morph samples were available for each tissue. Accession numbers of each sample used in this study can be found in Supplementary Tables S1, S3 and S4.

### Quantification of gene expression

Reads of each RNA-seq sample files were trimmed using Trimmomatic^65^ v0.38, with the following parameters: ILLUMINACLIP:2:30:10 SLIDINGWINDOW:4:5 LEADING:5 TRAILING:5 MINLEN:25. Trimmed reads were then used to run Salmon^66^ v1.4.0 ‘quant’ (mapping-based mode) to quantify gene expression (–gcBias –validateMappings). *P. veris* and *P. vulgaris* RNA-seq reads were mapped against the *P. veris* coding sequences^17^, while *F. esculentum* RNA-seq reads were mapped against the *F. esculentum* coding sequences from ref. ^46^.

To build the *P. veris* transcriptome atlas, first the Salmon output (i.e. read counts in the 20 *P. veris* RNA-seq samples for each of the 34,441 gene) was imported into R v3.6.3 (https://www.R-project.org/) using tximport^67,68^ and a DESeqDataSet was created with the DESeqDataSetFromTximport function of DESeq2^69^ (R/Bioconductor^70^ package). In importing transcript quantifications into DESeq2, we summarized expression at the gene level. Genes showing zero counts in all samples were removed, leaving with a total of 31,112 genes whose counts were then normalized using the default median of ratios method^69^. An “expression per tissue” matrix was created by calculating the average among the normalized per-gene counts of the two/three samples for each tissue. The normalized counts per sample were log_2_ transformed and their distribution plotted (Suppl. Fig. S1); the resulting plot showed a main distribution centered at a value of ~ 10 log_2_ counts (~ 1024 counts) with a shoulder at the left of this distribution. Such a bimodal distribution of gene expression is often observed^35^ and can be used to discriminate transcriptionally active genes (main distribution) from low-expression genes (left shoulder of the distribution)^35^. Based on this distribution we selected a threshold of 2 log_2_ counts (~ 4 counts) to classify a gene as transcriptionally active (≥ 4 counts) or as not expressed (< 4 counts).

### Differential gene expression analysis between floral and non-floral tissues

The 20 *P. veris* RNA-seq samples from BioProject PRJEB44353 were used also to compare the expression of *S-*locus genes and their paralogs between floral tissues (floral buds, flower) and non-floral tissues (root, seed, seedling, leaf, inflorescence stem). In brief, each RNA-seq sample was defined as “floral” or “non-floral” depending on its tissue of origin and imported into DESeq2 as described above. Then the DESeq function of DESeq2 was run with default parameters (false discovery rate controlled using the Benjamini–Hochberg method) to identify DEGs between floral and non-floral tissues.

### Differential gene expression analysis between L- and S-morphs

Differential gene expression analysis between L- and S-morphs was performed for two *P. veris* samples (style and corolla tube), one *P. vulgaris* samples (whole flower) and three *F. esculentum* samples (stamen filament, young carpel, mature carpel) using DESeq2. For all RNA-seq samples, reads were trimmed, gene expression quantified and counts imported into DESeq2 as described above. The identification of DEGs was then carried out using the DESeq function of DESeq2 with default parameters: false discovery rate was controlled using the Benjamini–Hochberg method and a filter was then applied to exclude genes showing log_2_-fold between -1 and 1, and adjusted p-value (padj) > 0.05.

### Identification of *F. esculentum* S-locus-related genes

Sequences for *S-ELF3* (GenBank accession: AB642167), SSG2 (AB668598), and PG1 (Buckwheat Genome Data Base^19^ gene ID: Fes_sc0006922.1. g000006.aua.1) were translated and searched against the translated CDS of *F. esculentum* (from ref. ^46^) using Proteinortho^71^ v6.0.31 (-p = blastp -e = 1e-5 -sim = 1).

### Functional annotation of *P. veris* and *F. esculentum* genes

The functional annotation of *P. veris* and *F. esculentum* genes was performed in two ways. First, 8,437 and 8,380 gene ontology (GO) terms were assigned to 23,673 and 23,516 genes of *P. veris* and *F. esculentum*, respectively, using TRAPID^72^ v2.0 (http://bioinformatics.psb.ugent.be/trapid_02). Second, one-to-one *A. thaliana*^73^ (TAIR10) orthologs were identified for 10,872 and 9,659 *P. veris and F. esculentum* genes, respectively using Proteinortho^71^ v6.0.31 (-p = blastp -e = 1e-5 -sim = 1), to aid functional description of the genes. To perform GO enrichment analyses on the DEGs identified in *P. veris* and *F. esculentum*, we used the ‘enricher’ function of the clusterProfiler v3.14.3 package^74^ (pvalueCutoff = 0.05, pAdjustMethod = BH, qvalueCutoff = 0.2).

### Gene orthology and comparative transcriptomics analyses

DEGs were identified in the present study between L- and S-morphs in *Primula* and *F. esculentum* (see above). A list of DEGs between L- and S-morphs identified in four floral tissues of *T. subulata* (young pistil, mature pistil, young stamen, mature stamen) had already been generated in a previous study^22^. We performed an analysis to identify orthologous and paralogous genes using OrthoFinder^75,76^ v2.3.11 (-I 1.7) on the proteomes of the three distylous species studied here (*P. veris*, *F. esculentum*, *T. subulata*) and nine other angiosperm species selected to be closely-related to the distylous species mentioned above (*Fagopyrum tataricum*, *Passiflora edulis*) or to have high-quality and well-annotated proteomes (*Antirrhinum majus*, *Arabidopsis thaliana*, *Daucus carota*, *Medicago truncatula*, *Prunus persica*, *Solanum lycopersicum*, *Vitis vinifera*). In summary, 359,400 genes (out of 408,413; 88%) were assigned to 32,436 orthogroups, (mean number of genes per orthogroup: 11.1). We then investigated whether any orthogroup contained DEGs identified in all the three species, analyzing separately male and female organs and *S-*locus up- and down- regulated genes (four analyses in total).

Phylogenetic reconstruction was conducted for OG0000060 orthogroup and for OG0000315 orthogroup plus other FMO sequences from *A. thaliana* and *H. vulgare*^51^. Sequences were aligned with MAFFT^77^ using global pairwise alignment (–globalpair) and a maximum of 1000 iterations (–maxiterate 1000). For the analysis of the OG0000060 orthogroup, seven sequences (DCAR_003205, FtPinG0505021700.01.T01, FtPinG0505025300.01.T01, FtPinG0505414500.01.T01, Solyc10g052570.1.1, tr_4883, tr_8798) were removed after being identified as having ambiguous or insufficient phylogenetic signal (complete phylogeny in Suppl. Fig. S5). Maximum Likelihood (ML) phylogenetic trees were constructed using IQ-TREE^78^ v2.1.2. For each alignment, the best protein model was selected by ModelFinder^79^ and compared by Bayesian Information Criterion (BIC). Branch support was assessed by 1,000 ultrafast bootstrap replicates^80^.

### PIF enrichment analysis

A total of 9,122 one-to-one *A. thaliana* orthologs were identified for *T. subulata* in the same way described for *P. veris* and *F. esculentum*. Each gene of the three distylous species with an *A. thaliana* ortholog was marked as “PIF-regulated” or “non-PIF-regulated” based on a list of 1,070 *A. thaliana* genes annotated as PIF-regulated obtained from a previous study^32^. For each sample of each species a contingency table containing the number of PIF-regulated and non-PIF-regulated for both DEGs and non-DEGs was built and a Fisher’s exact test was applied.

## Supplementary Information

Supplementary Figures.

Supplementary Tables.

## Data Availability

The datasets analysed during the current study are available in the NCBI repository (https://www.ncbi.nlm.nih.gov/) under the BioProject IDs PRJEB44353 (https://www.ncbi.nlm.nih.gov/bioproject/?term=PRJEB44353), PRJNA317964 (https://www.ncbi.nlm.nih.gov/bioproject/?term=PRJNA317964), PRJEB9683 (https://www.ncbi.nlm.nih.gov/bioproject/?term=PRJEB9683), and PRJNA487842 (https://www.ncbi.nlm.nih.gov/bioproject/?term=PRJNA487842). For details, see Methods.

## References

[1] Barrett SCH (2002). The evolution of plant sexual diversity. Nat. Rev. Genet..

[2] Darwin, C. *The Different Forms of Flowers on Plants of the Same Species*. (Murray, 1877).

[3] Shivanna KR, Heslop-Harrison J, Heslop-Harrison Y (1981). Heterostyly in Primula. 2. Sites of pollen inhibition, and effects of pistil constituents on compatible and incompatible pollen-tube growth. Protoplasma.

[4] Richards, J. H. & Barrett, S. C. H. The Development of Heterostyly. in *Evolution and function of heterostyly. Monographs on Theoretical and Applied Genetics* (ed. Barrett, S. C. H.) 85–128 (Springer, 1992). 10.1007/978-3-642-86656-2_4.

[5] Heslop-Harrison Y, Heslop-Harrison J, Shivanna KR (1981). Heterostyly in Primula. 1. Fine-structural and cytochemical features of the stigma and style in *Primula vulgaris* huds. Protoplasma.

[6] Piper J, Charlesworth B (1986). The evolution of distyly in *Primula vulgaris*. Biol. J. Linn. Soc..

[7] Ganders FR (1979). The biology of heterostyly. New Zeal. J. Bot..

[8] Webster MA, Gilmartin PM (2006). Analysis of late stage flower development in *Primula vulgaris* reveals novel differences in cell morphology and temporal aspects of floral heteromorphy. New Phytol..

[9] Naiki A (2012). Heterostyly and the possibility of its breakdown by polyploidization. Plant Species Biol..

[10] Barrett SCH (2019). ‘A most complex marriage arrangement’: recent advances on heterostyly and unresolved questions. New Phytol..

[11] Nowak MD (2015). The draft genome of *Primula veris* yields insights into the molecular basis of heterostyly. Genome Biol..

[12] Li J (2016). Genetic architecture and evolution of the *S* locus supergene in *Primula vulgaris*. Nat. Plants.

[13] Huu CN (2016). Presence versus absence of *CYP734A50* underlies the style-length dimorphism in primroses. Elife.

[14] Cocker JM (2018). *Primula vulgaris* (primrose) genome assembly, annotation and gene expression, with comparative genomics on the heterostyly supergene. Sci. Rep..

[15] Huu CN, Keller B, Conti E, Kappel C, Lenhard M (2020). Supergene evolution via stepwise duplications and neofunctionalization of a floral-organ identity gene. Proc. Natl. Acad. Sci. U. S. A..

[16] Huu CN, Plaschil S, Himmelbach A, Kappel C, Lenhard M (2022). Female self-incompatibility type in heterostylous *Primula* is determined by the brassinosteroid-inactivating cytochrome P450 *CYP734A50*. Curr. Biol..

[17] Potente G (2022). Comparative Genomics elucidates the origin of a supergene controlling floral heteromorphism. Mol. Biol. Evol..

[18] Yasui Y (2012). *S-LOCUS EARLY FLOWERING&nbsp;3* is exclusively present in the genomes of short-styled buckwheat plants that exhibit heteromorphic self-incompatibility. PLoS ONE.

[19] Yasui Y (2016). Assembly of the draft genome of buckwheat and its applications in identifying agronomically useful genes. DNA Res..

[20] Shore JS (2019). The long and short of the S-locus in *Turnera* (Passifloraceae). New Phytol..

[21] Matzke CM, Shore JS, Neff MM, McCubbin AG (2020). The *Turnera* style *S*-locus gene *TsBAHD* possesses brassinosteroid-inactivating activity when expressed in *Arabidopsis thaliana*. Plants.

[22] Henning PM, Shore JS, McCubbin AG (2020). Transcriptome and network analyses of heterostyly in *Turnera subulata* provide mechanistic insights: Are *S*-loci a red-light for pistil elongation?. Plants.

[23] Henning PM, Shore JS, McCubbin AG (2022). The *S*-Gene *YUC6* pleiotropically determines male mating type and pollen size in heterostylous *Turnera* (Passifloraceae): A novel neofunctionalization of the *YUCCA* Gene Family. Plants.

[24] Gutiérrez-Valencia J (2022). Genomic analyses of the *Linum* distyly supergene reveal convergent evolution at the molecular level. Curr. Biol..

[25] Kappel C, Huu CN, Lenhard M (2017). A short story gets longer: Recent insights into the molecular basis of heterostyly. J. Exp. Bot..

[26] Cohen JI (2016). *De novo* sequencing and comparative transcriptomics of floral development of the distylous species *Lithospermum multiflorum*. Front. Plant Sci..

[27] Hayta S, Smedley MA, Li J, Harwood WA, Gilmartin PM (2016). Plant regeneration from leaf-derived callus cultures of primrose (*Primula vulgaris*). HortScience.

[28] Ohnishi T (2006). Tomato cytochrome P450 CYP734A7 functions in brassinosteroid catabolism. Phytochemistry.

[29] Matsui K, Mizuno N, Ueno M, Takeshima R, Yasui Y (2020). Development of co-dominant markers linked to a hemizygous region that is related to the self-compatibility locus (*S*) in buckwheat (*Fagopyrum esculentum*). Breed. Sci..

[30] Urban, I. *Monographie der familie der Turneraceen*. (Gebruder Borntraeger, 1883).

[31] Matzke CM (2021). Pistil mating type and morphology are mediated by the brassinosteroid inactivating activity of the *S*-Locus gene *BAHD* in Heterostylous *Turnera* Species. Int. J. Mol. Sci..

[32] Leivar P, Monte E (2014). PIFs: Systems integrators in plant development. Plant Cell.

[33] Weinig C (2002). Phytochrome photoreceptors mediate plasticity to light quality in flowers of the Brassicaceae. Am. J. Bot..

[34] Hebenstreit D (2011). RNA sequencing reveals two major classes of gene expression levels in metazoan cells. Mol. Syst. Biol..

[35] Hart T, Komori HK, LaMere S, Podshivalova K, Salomon DR (2013). Finding the active genes in deep RNA-seq gene expression studies. BMC Genomics.

[36] Klepikova AV, Penin AA (2019). Gene expression maps in plants: Current state and prospects. Plants.

[37] Yanai I (2005). Genome-wide midrange transcription profiles reveal expression level relationships in human tissue specification. Bioinformatics.

[38] Kozera B, Rapacz M (2013). Reference genes in real-time PCR. J. Appl. Genet..

[39] Zhiponova MK (2014). Helix-loop-helix/basic helix-loop-helix transcription factor network represses cell elongation in *Arabidopsis* through an apparent incoherent feed-forward loop. Proc. Natl. Acad. Sci. U. S. A..

[40] Castellano MM, del Pozo JC, Ramirez-Parra E, Brown S, Gutierrez C (2001). Expression and stability of arabidopsis *CDC6* are associated with endoreplication. Plant Cell.

[41] Vandepoele K (2005). Genome-wide identification of potential plant E2F target genes. Plant Physiol..

[42] Gutierrez C (2009). The arabidopsis cell division cycle. Arab. B..

[43] Ranocha P (2013). Arabidopsis WAT1 is a vacuolar auxin transport facilitator required for auxin homoeostasis. Nat. Commun..

[44] Wang L, Ruan YL (2013). Regulation of cell division and expansion by sugar and auxin signaling. Front. Plant Sci..

[45] Takeshima R, Nishio T, Komatsu S, Kurauchi N, Matsui K (2019). Identification of a gene encoding polygalacturonase expressed specifically in short styles in distylous common buckwheat (*Fagopyrum esculentum*). Hered..

[46] Penin AA (2021). High-resolution transcriptome atlas and improved genome assembly of common buckwheat. Fagopyrum esculentum. Front. Plant Sci..

[47] Chae K (2012). Arabidopsis *SMALL AUXIN UP RNA63* promotes hypocotyl and stamen filament elongation. Plant J..

[48] Spartz AK (2014). SAUR inhibition of PP2C-D phosphatases activates plasma membrane H+-ATPases to promote cell expansion in Arabidopsis. Plant Cell.

[49] van Berkel WJH, Kamerbeek NM, Fraaije MW (2006). Flavoprotein monooxygenases, a diverse class of oxidative biocatalysts. J. Biotechnol..

[50] Cheng Y, Dai X, Zhao Y (2006). Auxin biosynthesis by the *YUCCA* flavin monooxygenases controls the formation of floral organs and vascular tissues in *Arabidopsis*. Genes Dev..

[51] Thodberg S, Neilson EHJ (2020). The, “green” FMOs: Diversity, functionality and application of plant flavoproteins. Catalysts.

[52] Hansen BG, Kliebenstein DJ, Halkier BA (2007). Identification of a flavin-monooxygenase as the S-oxygenating enzyme in aliphatic glucosinolate biosynthesis in Arabidopsis. Plant J..

[53] Rhodes J (2022). Perception of a conserved family of plant signalling peptides by the receptor kinase HSL3. Elife.

[54] He K (2007). BAK1 and BKK1 regulate brassinosteroid-dependent growth and brassinosteroid-independent cell-death pathways. Curr. Biol..

[55] Planas-Riverola A (2019). Brassinosteroid signaling in plant development and adaptation to stress. Development.

[56] Maurel C, Verdoucq L, Luu DT, Santoni V (2008). Plant aquaporins: Membrane channels with multiple integrated functions. Annu. Rev. Plant Biol..

[57] Maurel C (2015). Aquaporins in plants. Physiol. Rev..

[58] Wang Y, Zhao Z, Liu F, Sun L, Hao F (2020). Versatile roles of aquaporins in plant growth and development. Int. J. Mol. Sci..

[59] Ludevid D, Höfte H, Himelblau E, Chrispeels MJ (1992). The expression pattern of the tonoplast intrinsic protein γ-TIP in Arabidopsis thaliana Is correlated with cell enlargement. Plant Physiol..

[60] Ma N (2008). *Rh-PIP2;1*, a rose aquaporin gene, is involved in ethylene-regulated petal expansion. Plant Physiol..

[61] Aharon R (2003). Overexpression of a plasma membrane aquaporin in transgenic tobacco improves plant vigor under favorable growth conditions but not under drought or salt stress. Plant Cell.

[62] Warner MR, Qiu L, Holmes MJ, Mikheyev AS, Linksvayer TA (2019). Convergent eusocial evolution is based on a shared reproductive groundplan plus lineage-specific plastic genes. Nat. Commun..

[63] Washburn JD, Bird KA, Conant GC, Pires JC (2016). Convergent evolution and the origin of complex phenotypes in the age of systems biology. Int. J. Plant Sci..

[64] Stortenbeker N, Bemer M (2019). The *SAUR* gene family: The plant’s toolbox for adaptation of growth and development. J. Exp. Bot..

[65] Bolger AM, Lohse M, Usadel B (2014). Trimmomatic: A flexible trimmer for Illumina sequence data. Bioinformatics.

[66] Patro R, Duggal G, Love MI, Irizarry RA, Kingsford C (2017). Salmon: fast and bias-aware quantification of transcript expression using dual-phase inference. Nat. Methods.

[67] Soneson C, Love MI, Robinson MD (2016). Differential analyses for RNA-seq: transcript-level estimates improve gene-level inferences. F1000Research.

[68] Love MI, Soneson C, Patro R, Vitting-Seerup K, Thodberg M (2018). Swimming downstream: statistical analysis of differential transcript usage following Salmon quantification. F1000Research.

[69] Love MI, Huber W, Anders S (2014). Moderated estimation of fold change and dispersion for RNA-seq data with DESeq2. Genome Biol..

[70] Gentleman RC (2004). Bioconductor: Open software development for computational biology and bioinformatics. Genome Biol..

[71] Lechner M (2011). Proteinortho: Detection of (Co-)orthologs in large-scale analysis. BMC Bioinf..

[72] van Bel M (2013). TRAPID: An efficient online tool for the functional and comparative analysis of *de novo* RNA-Seq transcriptomes. Genome Biol..

[73] Lamesch P (2012). The Arabidopsis Information Resource (TAIR): improved gene annotation and new tools. Nucl. Acids Res..

[74] Yu G, Wang LG, Han Y, He QY (2012). ClusterProfiler: An R package for comparing biological themes among gene clusters. Omi. A J. Integr. Biol..

[75] Emms DM, Kelly S (2015). OrthoFinder: Solving fundamental biases in whole genome comparisons dramatically improves orthogroup inference accuracy. Genome Biol..

[76] Emms DM, Kelly S (2019). OrthoFinder: Phylogenetic orthology inference for comparative genomics. Genome Biol..

[77] Katoh K, Standley DM (2013). MAFFT multiple sequence alignment software version 7: Improvements in performance and usability. Mol. Biol. Evol..

[78] Nguyen LT, Schmidt HA, Von Haeseler A, Minh BQ (2015). IQ-TREE: A fast and effective stochastic algorithm for estimating maximum-likelihood phylogenies. Mol. Biol. Evol..

[79] Kalyaanamoorthy S, Minh BQ, Wong TKF, Von Haeseler A, Jermiin LS (2017). ModelFinder: Fast model selection for accurate phylogenetic estimates. Nat. Methods.

[80] Minh BQ, Nguyen MAT, Von Haeseler A (2013). Ultrafast approximation for phylogenetic bootstrap. Mol. Biol. Evol..

