# Supplementary material for: Comparative transcriptomics reveals commonalities and differences in the genetic underpinnings of a floral dimorphism
Source: Sci Rep. 2022 Dec 1;12:20771. doi: 10.1038/s41598-022-25132-2 (PMC9715534; doi:10.1038/s41598-022-25132-2)
Supplement: Supplementary file 1 — Supplementary Figures. [file 41598_2022_25132_MOESM1_ESM.pdf]

Supplementary Figures for *"Comparative transcriptomics reveals commonalities and differences in the genetic underpinnings of a floral dimorphism"*

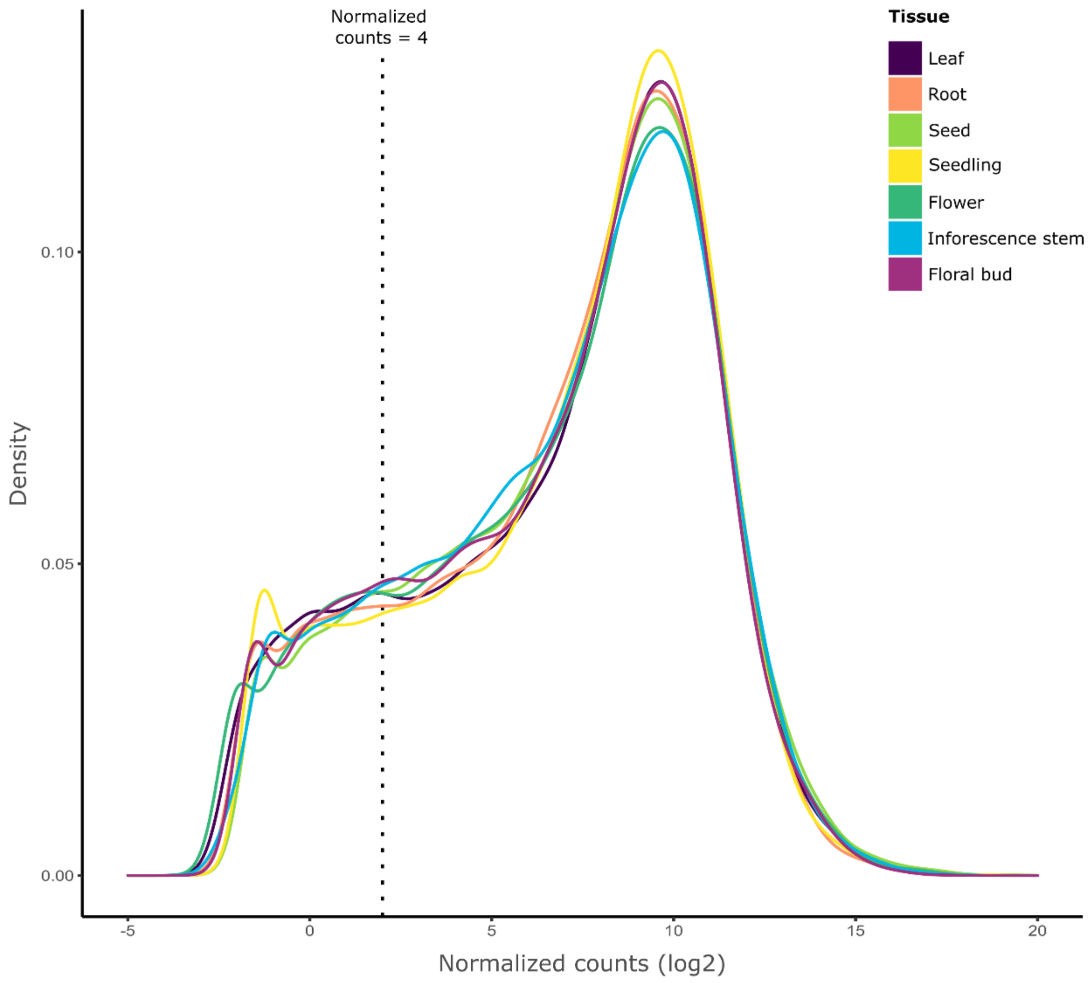

Supplementary Figure 1. Distribution of gene expression across all tissues. Density plot representing the distributions of normalized read counts for genes expressed in the seven tissues analyzed, each colored as shown in the legend. A dotted vertical line at log2(normalized counts) = 2 (i.e. normalized counts = 4) indicates the threshold used to mark a gene as expressed.

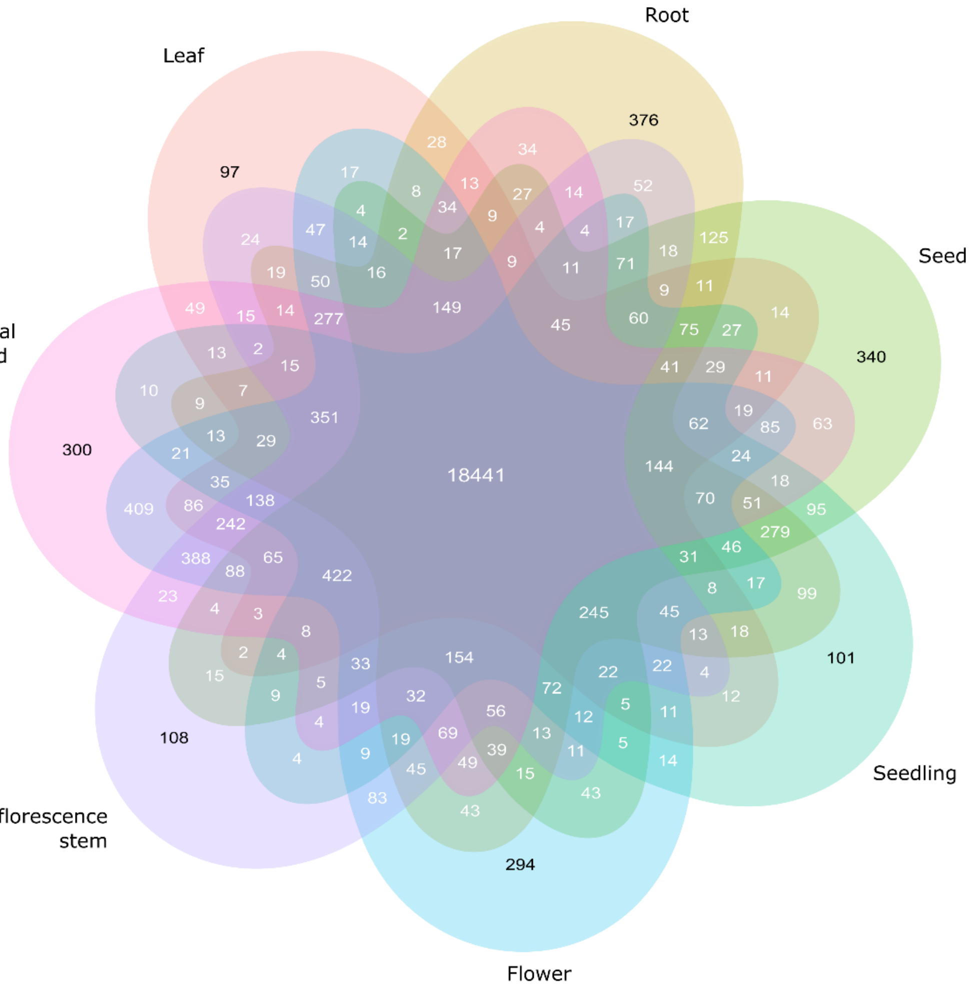

Supplementary Figure S2: Venn diagram with the number of genes expressed in each tissue.

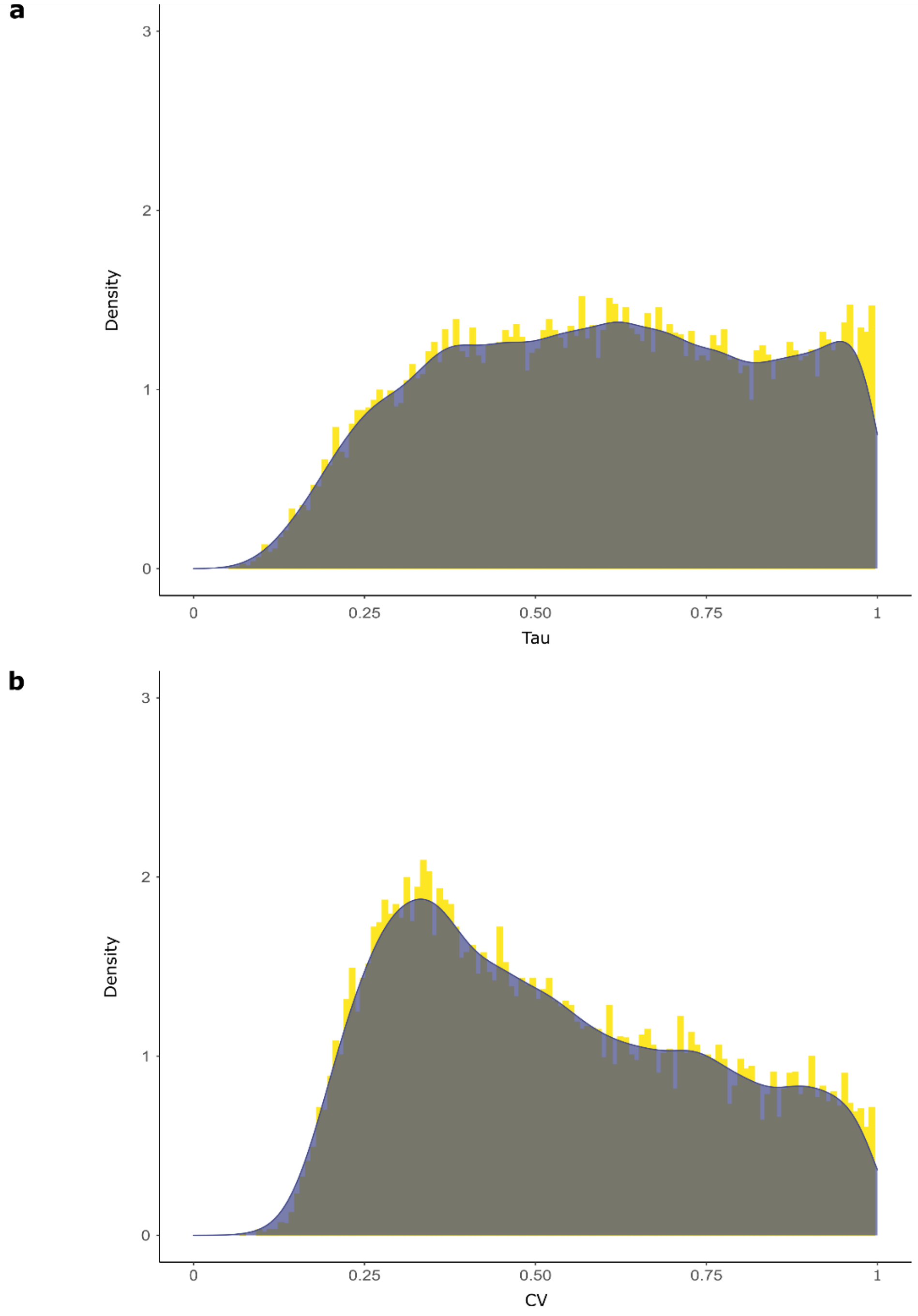

Supplementary Figure S3: Distribution of tissue-specificity for the *P. veris* gene set. a. Distribution of the tau tissue-specificity index. **b.** Distribution of the coefficient of variation (CV).

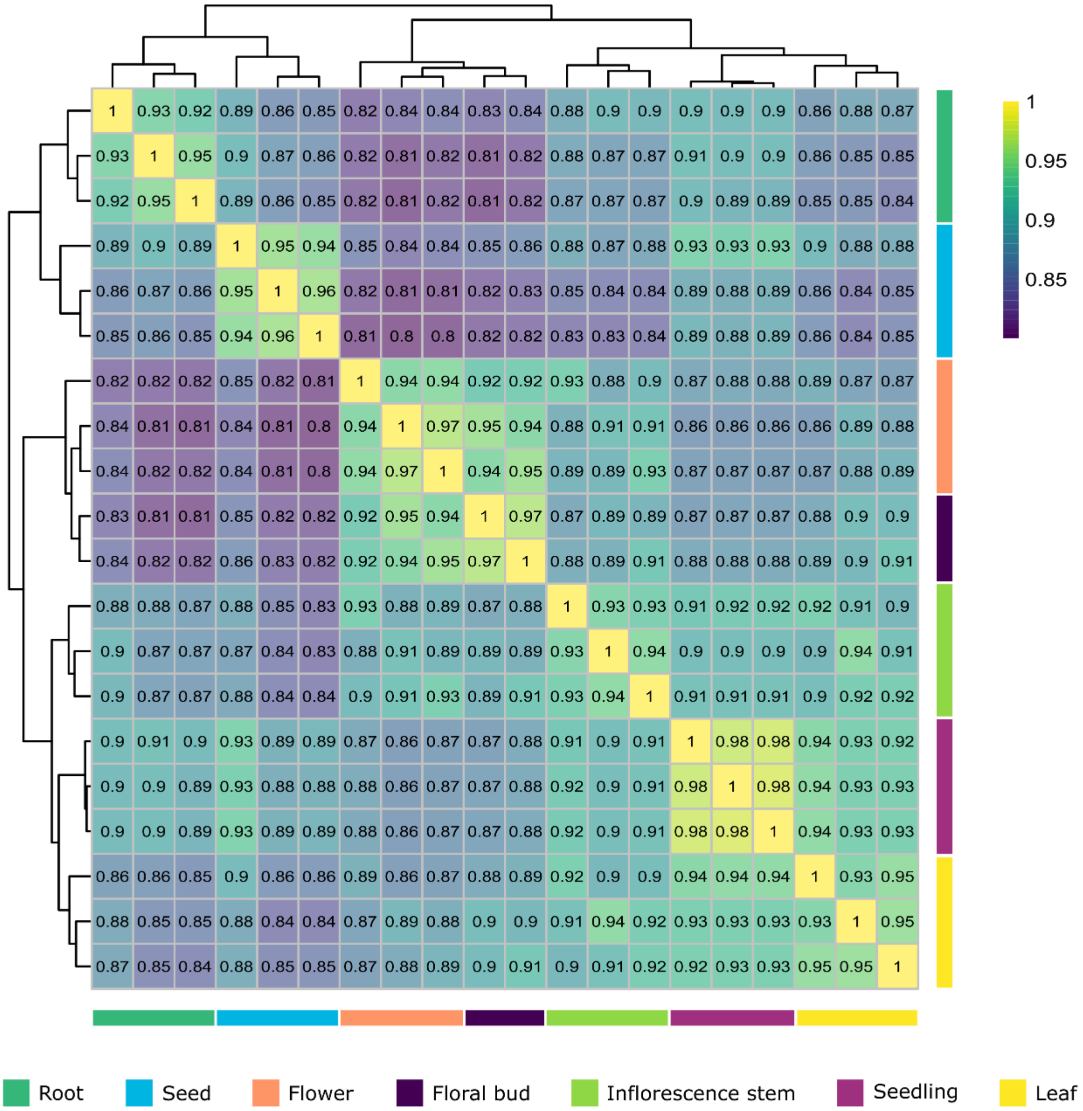

Supplementary Figure S4: Heatmap of hierarchical clustering of all 20 samples. Heatmap of hierarchical clustering of the samples based on their pairwise similarity (estimated as Spearman's rank correlations) for all 20 samples. This figure is the same as Fig. 1b of the main text, but here the degree of correlation between samples is written in each cell, in addition to being indicated by color scale. Samples of the same tissue are indicated by the same color bar on the sides.

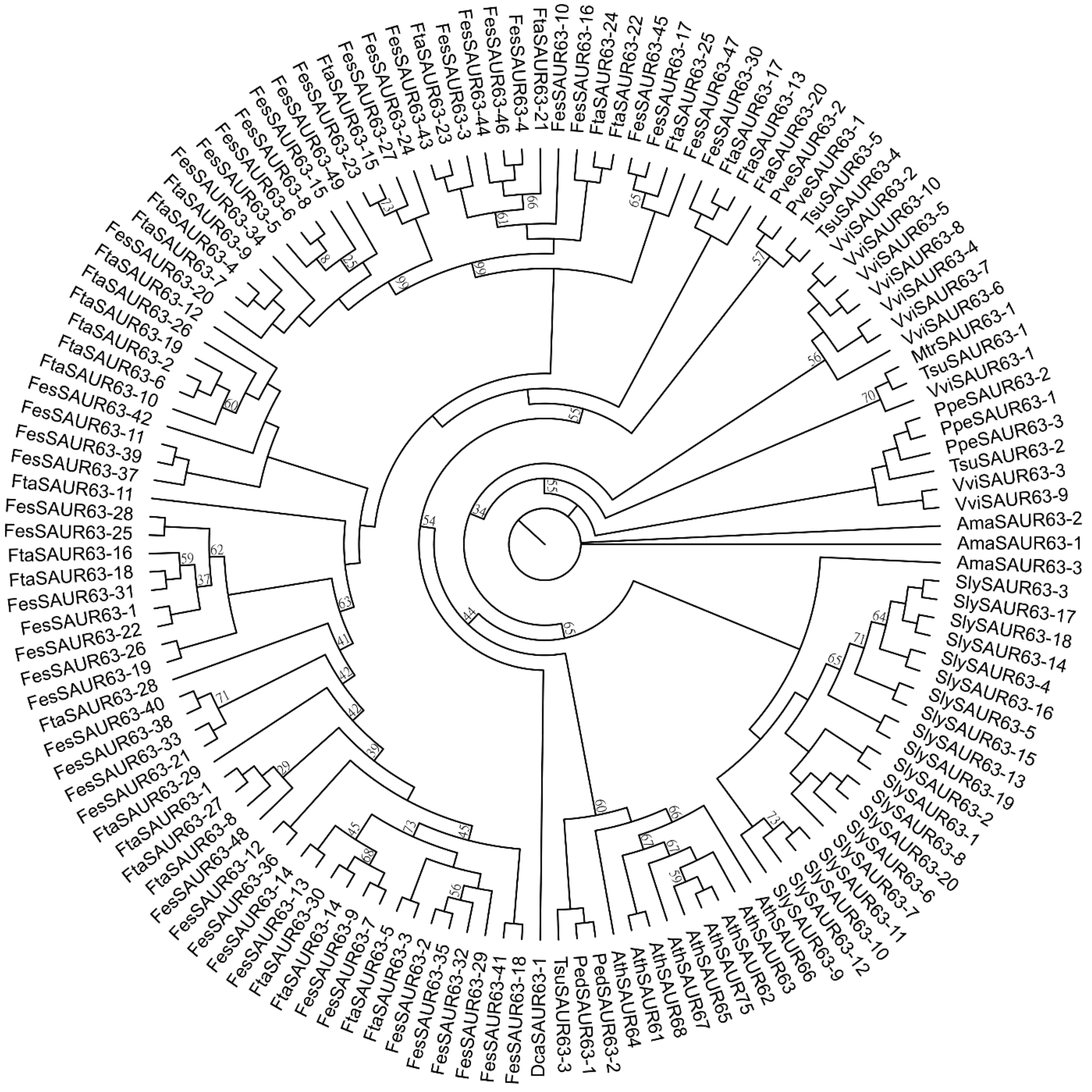

Supplementary Figure S5: Complete phylogeny of the OG0000060 orthogroup. Bootstrap values <75 are shown. Compared to the OG0000060 orthogroup phylogeny shown in Fig. 6e, here also the sequences of DCAR_003205 (DcaSAUR63-1), FtPinG0505021700.01.T01 (FtaSAUR63-29), FtPinG0505025300.01.T01 (FtaSAUR63-30), FtPinG0505414500.01.T01 (FtaSAUR63-28), Solyc10g052570.1.1 (SlySAUR63-20), tr_4883 (FesSAUR63-49), and tr_8798 (FesSAUR63-48) are included.
